# Angiotensin-Converting Enzyme 2 Overexpression Protects Heart from Aging-Induced Injury in C57BL/6 Mice

**DOI:** 10.3390/ijms27115082

**Published:** 2026-06-04

**Authors:** Chunyan Chen, Na Sun, Hanyue Zheng, Han Zhang, Lin Miao

**Affiliations:** 1 Key Laboratory of Pharmacology of Traditional Chinese Medical Formulae, Ministry of Education, Tianjin University of Traditional Chinese Medicine, Tianjin 301617, China; 18848820783@163.com (C.C.);; 2 State Key Laboratory of Chinese Medicine Modernization, Tianjin University of Traditional Chinese Medicine, Tianjin 301617, China; 3 State Key Laboratory of Component-Based Chinese Medicine, Tianjin University of Traditional Chinese Medicine, Tianjin 301617, China

**Keywords:** angiotensin-converting enzyme 2, cardiac aging, renin–angiotensin system, angiotensin II, cardiovascular disease

## Abstract

Cardiovascular disease (CVD) is a leading cause of morbidity and mortality globally among older adults. Similar to humans, age-related declines in cardiac function are observed in C57BL/6 mice. Angiotensin-converting enzyme 2 (ACE2), a key component of the renin–angiotensin system (RAS), counteracts detrimental RAS effects by converting angiotensin II (Ang II) to angiotensin-(1-7) (Ang-(1-7)), thereby playing a critical role in mitigating CVD pathogenesis. Here, we utilized transgenic K18-hACE2 mice to investigate the protective effects of ACE2 against cardiac aging. Histological and morphometric analyses revealed significant reductions in heart weight and improvements in cardiac structure in K18-hACE2 mice compared to wild-type controls. Furthermore, aged C57BL/6 mice exhibited progressive cardiac aging phenotypes, including mitochondrial dysfunction, telomere shortening, and immune dysregulation—all of which were significantly attenuated in K18-hACE2 mice. These findings demonstrate the protective role of ACE2 in cardiac aging and highlight its potential as a therapeutic target for anti-aging interventions.

## 1. Introduction

Aging is a major risk factor for cardiovascular disease (CVD) in older adults [1]. Globally, an estimated 17.9 million people die from CVD annually, accounting for 31% of all global deaths [1]. Among individuals over 50 years of age, approximately 1% experience heart failure, with the prevalence doubling every subsequent decade [2]. Cardiac aging manifests as progressive declines in cardiac structure and function, including ventricular hypertrophy, left ventricular dysfunction, atrial remodeling, and increased fibrosis [3,4]. These age-related degenerative changes are frequently accompanied by elevated incidence rates of hypertension [5], atherosclerosis [6], and heart failure [7], significantly compromising both healthspan and lifespan. Therefore, elucidating the molecular mechanisms underlying cardiac aging is essential for developing novel preventive and therapeutic strategies.

ACE2 is a monocarboxypeptidase widely expressed in cardiomyocytes, cardio fibroblasts, and coronary endothelial cells [8,9,10,11]. Functionally, ACE2 converts angiotensin II (Ang II) into angiotensin-(1-7) (Ang (1-7)), thereby counteracting the molecular and cellular effects of Ang II [12,13]. Structurally, as a type I transmembrane protein, ACE2 undergoes proteolytic cleavage by proteases [12], leading to ectodomain shedding from the cell membrane. This shedding reduces membrane-bound ACE2 expression, impairing its cardioprotective effects and exacerbating cardiac fibrosis [14,15]. As a key negative regulator of the renin–angiotensin system (RAS), ACE2 deletion aggravates myocardial injury and ventricular remodeling in heart failure and dilated cardiomyopathy [16,17]. Conversely, gain-of-function interventions, including recombinant human ACE2 (rhACE2) administration, ACE2 overexpression, or Ang-(1-7) supplementation, demonstrate protective effects in CVD models such as hypertension, diabetes, and heart failure [16,18,19,20].

Based on the hallmarks of aging framework [21,22], eight core characteristics of cardiac aging have been identified: cellular senescence, mitochondrial dysfunction, disabled macro-autophagy, loss of proteostasis, genomic instability, epigenetic alterations, neurohormonal signaling disorders, and chronic inflammation [23]. These hallmarks collectively reflect biological age and cardiac integrity while synergistically driving aging progression. Notably, *ACE2* knockout has been demonstrated to accelerate progressive age-dependent dilated cardiomyopathy in mice at 6–12 months of age [17] and impair cerebral arterial endothelial function in 12–24-month-old mice [24]. Conversely, vascular ACE2 overexpression confers protection against age-related decline in renal function [20]. These findings suggest that ACE2 represents a potential therapeutic target for mitigating cardiovascular aging; however, mechanistic evidence linking ACE2 to specific cardiac aging hallmarks remains limited.

To investigate whether ACE2 delays cardiac aging and elucidate its underlying mechanisms, we assessed cardiac aging biomarkers and cardiac-specific markers in young and aged wild-type mice and K18-hACE2 transgenic mice (expressing human ACE2) on an isogenic genetic background.

## 2. Results

### 2.1. ACE2 Ameliorated Age-Related Cardiac Pathological Changes in Wild-Type Mice

**Figure 1:**
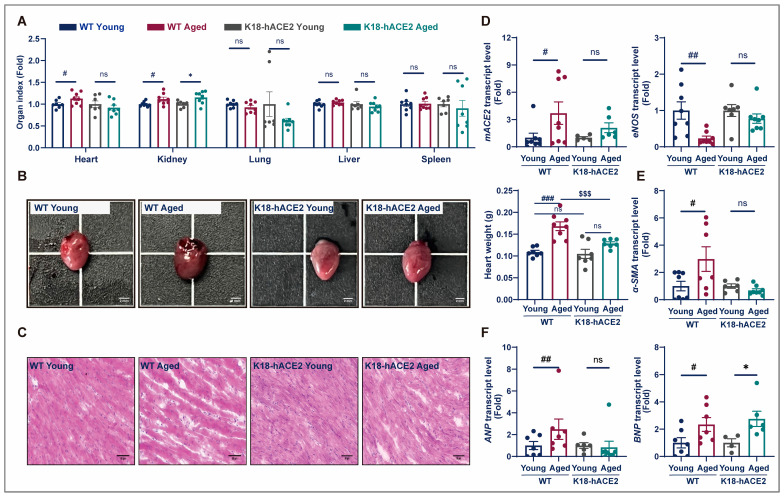
ACE2 alleviated the structural and functional abnormalities in the heart of aging mice. (**A**) Organ index in heart, kidney, lung, liver, and spleen (*n* = 7–8). (**B**) Representative images of hearts (scale bar, 2 mm, *n* = 3) and heart weight (*n* = 7–8). Statistical analysis of heart tissue weight presented in the figure above (*n* = 7–8). $$$ *p* < 0.001 compared with Aged WT group. (**C**) H&E staining of young and aged mice hearts. Scale bars, 50 μm (main images, *n* = 3). (**D**) Relative mRNA levels of m*ACE2* and *eNOS* in heart (*n* = 5–8). (**E**) Relative mRNA levels of *α-SMA* in heart (*n* = 6–8). (**F**) Relative mRNA levels of *ANP* and *BNP* in heart (*n* = 4–8). Results are expressed as mean ± SEM. # *p* < 0.05, ## *p* < 0.01 and ### *p* < 0.001 compared with Young WT group. * *p* < 0.05 compared with Young K18-hACE2 group.

Physiological aging induces systemic alterations in organ structure and function. Organ indices in aged mice were initially measured (Figure 1A). Heart and kidney indices were significantly increased in aged wild-type mice, whereas liver, lung, and spleen indices remained unaltered. Notably, aged K18-hACE2 transgenic mice demonstrated reduced heart weight, as evidenced by a lower heart index and smaller heart size compared to aged wild-type mice (Figure 1B).

Histological analysis revealed characteristic age-related changes in wild-type mice, including cardiomyocytes disarray—phenotypes that were substantially improved in K18-hACE2 mice (Figure 1C). To confirm the endogenous expression of ACE2 in the heart of mice, the mRNA levels of murine *ACE2* (m*ACE2*) were determined and data revealed an age-dependent upregulation of *mACE2* mRNA in wild-type mice, which was attenuated in K18-hACE2 mice (Figure 1D), suggesting feedback regulation of cardiac ACE2 expression. Furthermore, endothelial nitric oxide synthase (eNOS) expression, critical for cardiovascular homeostasis, declined in aged wild-type mice but remained stable in K18-hACE2 mice (Figure 1D). Atrial natriuretic peptide (ANP) and brain natriuretic peptide (BNP) are reliable biomarkers of myocardial volume load [25]. ANP and BNP mRNA levels were significantly upregulated with aging in wild-type mice; however, ACE2 overexpression attenuated ANP (but not BNP) upregulation (Figure 1F). Additionally, K18-hACE2 mice exhibited reduced expression of the fibrosis marker α-smooth muscle actin (α-SMA) compared to aged wild-type controls (Figure 1E).

### 2.2. ACE2 Mitigated Cardiac Aging Hallmarks in Wild-Type Mice

**Figure 2:**
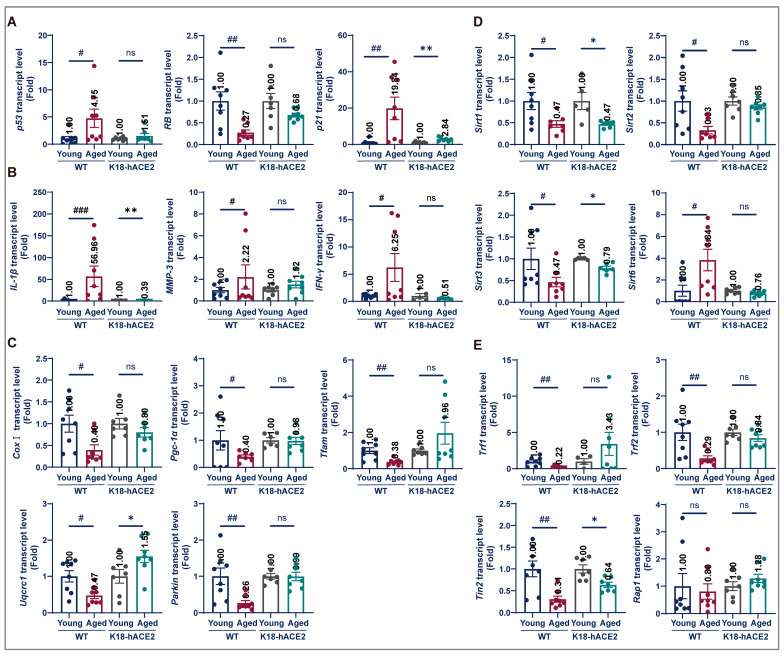
ACE2 ameliorated aging hallmarks in heart of aging mice. (**A**) Relative mRNA levels of cell cycle arrest factors *p53*, *RB*, and *p21* in heart (*n* = 7–8). (**B**) Relative mRNA levels of *IL-1β*, *MMP-3*, and *IFN-γ* in heart (*n* = 5–8). (**C**) Relative mRNA levels of mitochondrial dysfunction factors *Cox I*, *Pgc-1α*, *Tfam*, *Uqcrc1*, and *Parkin* in heart (*n* = 6–8). (**D**) Relative mRNA levels of *Sirt1*, *Sirt2*, *Sirt3*, and *Sirt6* in heart (*n* = 5–8). (**E**) Relative mRNA levels of *Trf1*, *Trf2*, *Tin2*, and *Rap1* in heart (*n* = 4–8). Results are expressed as mean ± SEM. # *p* < 0.05, ## *p* < 0.01 and ### *p* < 0.001 compared with Young WT group. * *p* < 0.05, and ** *p* < 0.01 compared with Young K18-hACE2 group.

As demonstrated in Figure 2A, elevated cardiac expression of senescence markers *p53* and *p21* was observed in aged wild-type mice, which was attenuated in K18-hACE2 mice. Retinoblastoma protein (RB), a transcription factor in the p53/p21 signaling pathway, declined significantly with aging in wild-type hearts but was preserved in K18-hACE2 mice. Pro-inflammatory mediators *interleukin-1 beta* (*IL-1β*), *matrix metalloproteinase-3* (*MMP-3*), and *interferon-gamma* (*IFN-γ*) were upregulated in aged wild-type hearts—a trend partially reversed by ACE2 overexpression (Figure 2B).

Mitochondrial dysfunction, a hallmark of cardiac aging, was evident in wild-type mice via downregulation of cytochrome c oxidase I (Cox I), peroxisome proliferator-activated receptor-gamma coactivator-1 alpha (Pgc-1α), mitochondrial transcription factor A (Tfam), and ubiquinol-cytochrome c reductase core protein 1 (Uqcrc1) mRNA (Figure 2C), which was restored in K18-hACE2 mice. Similarly, the mitophagy regulator parkin was suppressed in aged wild-type mice but maintained in K18-hACE2 mice.

Within the sirtuin family, *silent information regulator 2 homolog 1* (*Sirt1*) expression decreased significantly in both aged wild-type and K18-hACE2 mice. *Sirt2* and *Sirt3* declined dramatically with aging in wild-type hearts, but ACE2 overexpression attenuated these reductions (Figure 2D). Unexpectedly, *Sirt6* upregulated in aged wild-type mice but remained unchanged in K18-hACE2 mice.

Shelterin complex component telomeric repeat-binding factors 1 (*Trf1*), *Trf2* and *TRF1-interacting protein 2* (*Tin2*) decreased with aging in wild-type hearts, with attenuated declines in K18-hACE2 mice. *Rrepressor activator protein 1* (*Rap1*) mRNA was decreased in aged wild-type mice but trended upward in K18-hACE2 mice (Figure 2E).

Collectively, 12-month-old mice exhibited typical cardiac aging phenotypes—including cellular senescence, senescence-associated secretory phenotype (SASP), mitochondrial dysfunction, oxidative stress, and telomere attrition—all of which were ameliorated by ACE2, highlighting its role in delaying age-related cardiac degeneration.

### 2.3. ACE2 Restored Adaptive Immune Homeostasis in Aging Wild-Type Mice

**Figure 3:**
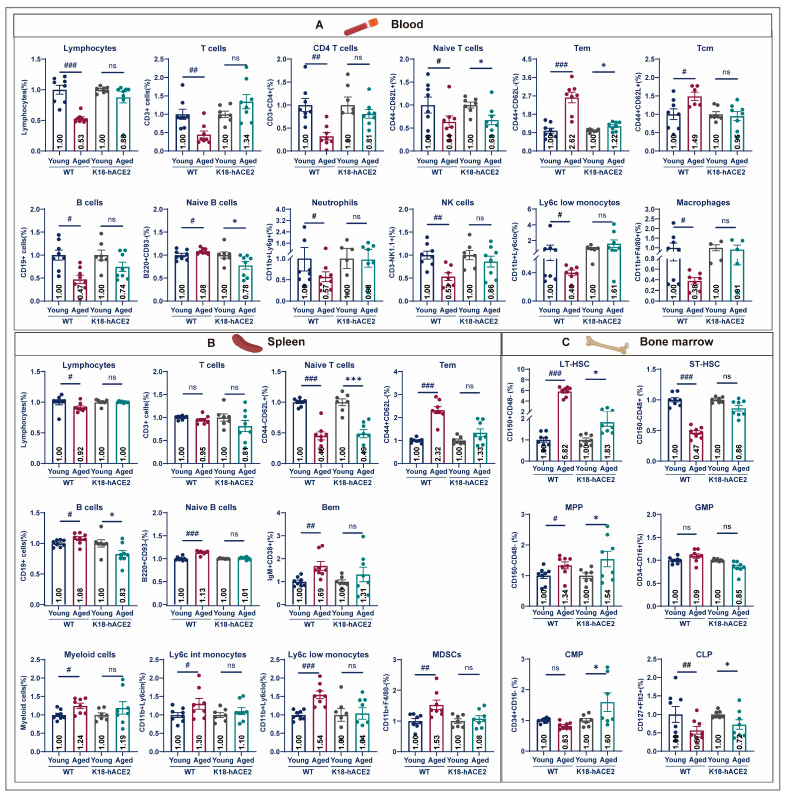
ACE2 improved the imbalance of immune cell subsets in aging mice. (**A**) Relative numbers of innate and adaptive immune cells in mouse blood (*n* = 4–8). (**B**) Relative numbers of innate and adaptive immune cells in mouse spleen (*n* = 7–8). (**C**) Relative numbers of LT-HSC, ST-HSC, MPP, GMP, CMP, and CLP in mouse bone marrow (*n* = 7–8). Results are expressed as mean ± SEM. # *p* < 0.05, ## *p* < 0.01 and ### *p* < 0.001 compared with Young WT group. * *p* < 0.05, and *** *p* < 0.001 compared with Young K18-hACE2 group.

Aging drives immune senescence, marked by systemic immune dysfunction. We analyzed immune cell subsets in peripheral blood, spleen, and bone marrow (Figure 3A–C).

In aged wild-type mice, lymphoid populations, including total T cells (CD3^+^), CD4^+^ T cells (CD3^+^CD4^+^), naive T cells (CD3^+^CD4^+^CD44^−^CD62L^+^), B cells (CD19^+^), and natural killer (NK, CD3^−^NK1.1^+^), declined markedly, while effector memory T cells (Tem, CD3^+^CD4^+^CD44^+^CD62L^−^), central memory T cells (Tcm, CD3^+^CD4^+^CD44^+^CD62L^+^), and naive B cells (CD19^+^B220^+^CD93^−^) increased. These age-related changes were attenuated in K18-hACE2 mice, with the exception of naive T cells (Figure 3A). Myeloid subsets including neutrophils (CD11b^+^Ly6g^+^), CD11b^+^Ly6c^low^ monocytes, and macrophages (CD11b^+^F4/80^+^) decreased in wild-type mice but remained stable in K18-hACE2 mice.

Similarly, in the spleen, aged wild-type mice exhibited reduced lymphoid cells and increased Tem, naive B, and Bem cells (CD19^+^B220^+^CD93^−^), which were reversed by ACE2 (Figure 3B). T cell diversity declined similarly in both aged groups. Notably, ACE2 not only reversed age-related B cell expansion but also mitigated myeloid cells, CD11b^+^Ly6c^int^ monocytes, CD11b^+^Ly6c^low^ monocytes, and myeloid-derived suppressor cells (MDSCs, CD11b^+^F4/80^−^) in wild-type mice.

Hematopoietic stem/progenitor cells (HSPCs) were perturbed with aging: long-term HSCs (LT-HSCs, Lin^-^c-kit^+^Sca-1^+^CD150^+^CD48^−^) increased in wild-type mice but only increased modestly in K18-hACE2 mice, while short-term HSCs (ST-HSCs, Lin^−^c-kit^+^Sca-1^+^CD150^+^CD48^+^) declined less severely in K18-hACE2 mice (Figure 3C). No significant changes occurred in multipotent progenitor cells (MPP, Lin^−^c-kit^+^Sca-1^+^CD150^−^CD48^−^) or granulocyte–monocyte progenitor cells (GMP, Lin^−^c-kit^+^Sca-1^−^CD34^+^CD16/32^+^) in both aged groups. Common lymphoid progenitor (CLP, Lin^−^c-kit^low^Sca-1^low^CD127^+^Flt3^+^) depletion in wild-type mice was partially rescued by ACE2, whereas common myeloid progenitors (CMP, Lin^-^c-kit^+^Sca-1^−^CD34^+^CD16/32^−^) increased exclusively in K18-hACE2 mice.

These findings indicate that ACE2 is involved in counteracting age-related adaptive immune imbalance and hematopoietic dysfunction.

## 3. Discussion

Recent discoveries have identified ACE2 as a critical therapeutic target in CVDs due to its central role in regulating angiotensin peptides. Our study demonstrated that aging C57BL/6 mice developed severe cardiomegaly, impaired cardiac systolic and diastolic function, and ventricular myocyte disorganization. Transgenic overexpression of ACE2 effectively mitigated these aging-induced alterations. Moreover, ACE2 ameliorated cardiac aging through the modulation of age-related gene expression patterns. Specifically, ACE2 downregulated the expression of cell cycle inhibitors (*p53*, *p21*, *RB*) and pro-inflammatory factors (*IL-1β*, *MMP-3*, *IFN-γ*). Conversely, ACE2 upregulated the expression of genes associated with mitochondrial biogenesis and function (*Cox I*, *Pgc-1α*, *Tfam*, *Uqcrc1*, *parkin*), sirtuin family members (*Sirt1*, *Sirt2*, *Sirt3*, *Sirt6*), and telomere-protective factors (*Trf*1, *Trf2*, *Tin2*, *Rap1*). Furthermore, ACE2 restored adaptive immune cell homeostasis in peripheral blood, spleen, and bone marrow. These findings, combined with previous evidence that *ACE2* deficiency exacerbates cardiomegaly and accelerates heart failure under pressure overload [16,17], highlighted its protective role in delaying cardiac aging.

Cardiac aging is characterized by structural alterations, which ultimately lead to impaired diastolic and systolic function [26]. Following transverse aortic constriction, ANP and BNP expression were significantly increased in *ACE2*-deficient mice when compared to wild-type mice [27], indicating a regulatory role for ACE2 in stress overload responses. Studies in murine and *Drosophila* models further demonstrated that *ACE2* disruption exacerbates stress-induced cardiac contractility defects [27,28]. In the present study, mice with transgenic ACE2 overexpression exhibited low levels of ANP (but not BNP), α-SMA in the heart and improved age-induced cardiac structure (Figure 1B,C,F), contrasting with reports of preserved ventricular function in aged *ACE2*-deficient mice [29]. Regarding the observed cardiac hypertrophy, obtaining definitive quantitative evidence requires measurements of the myocyte cross-sectional area using WGA or laminin staining. Furthermore, it has been found that with aging, the activity and expression of eNOS decline, which may increase CVD susceptibility [30]. Consistent with this, our study found that ACE2 ameliorated the significant decrease in *eNOS* expression in the hearts of wild-type mice (Figure 1D). Moreover, a previous study confirmed that ACE2 activated the eNOS pathway via the phosphatidylinositol 3-kinase (PI3K)/AKT-eNOS pathway and contributed to enhancing cardiac contractility [31], further supporting the relationship between ACE2 and eNOS.

Oudit et al. demonstrated that genetic *ACE2* deficiency exacerbated myocardial injury and promoted adverse ventricular remodeling in mice [17]. Notably, Ang II-induced cardiac injury was paradoxically associated with diminished cardiac ACE2 protein expression and enzymatic activity despite increased *ACE2* mRNA levels [14]. This apparent discordance between transcriptional and translational regulation contrasted sharply with clinical observations of elevated circulating ACE2 activity in patients with age-dependent heart failure [32]. Our results revealed that wild-type aging mice developed characteristic age-related cardiac pathologies. Strikingly, ACE2 overexpression attenuated these aging phenotypes while concomitantly suppressing endogenous ACE2 transcription (Figure 1D,E), suggesting a negative feedback loop regulating cardiac ACE2 expression, and the therapeutic potential of ACE2 augmentation for age-related cardiovascular dysfunction.

Cellular senescence, marked by irreversible cell cycle exit and proliferative arrest [2,33], is regulated by p53 and p21 [34,35]. While *ACE2* knockout mice exhibit age-dependent increases in p16^INK4a^ in skeletal muscle, they display no changes in *p21* or *p53* [36,37], indicating a unique aging-related pathway influenced by *ACE2* deficiency. Conversely, our study revealed that mice with ACE2 overexpression displayed reduced expression of cell cycle arrest markers (p53, p21) with age (Figure 2A), suggesting that ACE2 mitigates senescence by delaying cell cycle arrest. Senescent cells secrete pro-inflammatory factors like IL-1β (a hallmark of SASP), which drives inflammatory aging [2,38]. Paradoxically, IL-1β upregulated ACE2 expression in acute respiratory distress syndrome [39]. *ACE2* deficiency exacerbated vascular inflammation in Ang II-stimulated *ApoE*^−/−^ mice [10,40], whereas increased ACE2 expression reduced proinflammatory cytokines in sepsis-induced cardiomyopathy [41]. In our study, ACE2 overexpression downregulated cardiac *IL-1β*, *MMP-3*, and *IFN-γ* expression (Figure 2B), demonstrating its anti-inflammatory role in cardiac aging.

As one of the key aging mechanisms [26], mitochondrial dysfunction is modulated by ACE2. Intracellular ACE2 localizes to mitochondria in rodent and primate brains, reducing reactive oxygen species (ROS) [40]. Age-related decline in mitochondrial ACE2 impairs RAS-mediated protection [40]. Pharmacological ACE2 activation by diminazene aceturate (DIZE) enhances mitochondrial biogenesis via the MasR-SIRT1 pathway, whereas the ACE2 inhibitor (S,S)-2-(1-Carboxy-2-(3-(3,5-dichlorobenzyl)-3H-imidazol-4-yl)-ethylamino)-4-methylpentanoic acid (MLN-4760) exerts opposing effects [42]. Similarly, *ACE2^−/y^* mice exhibit impaired mitochondrial respiration and downregulated oxidative phosphorylation genes [43]. Consistent with these reports, our study demonstrated that K18-hACE2 mice preserved cardiac mitochondrial gene expression (*Cox I*, *Pgc-1α*, *Tfam*, *Uqcrc1*, *parkin*) compared to aged wild-type controls (Figure 2C), indicating ACE2’s role in preserving mitochondrial homeostasis during cardiac aging.

Sirtuins, NAD-dependent histone/protein deacetylases, regulate aging across species from yeast to mammals [44]. In our study, ACE2 overexpression restored sirtuin-mediated protection (Figure 2D), attenuating age-related cardiac decline. SIRT1, the most extensively studied mammalian sirtuin [45], improves age-related cardiac remodeling [46] and transcriptionally regulates ACE2 expression under energy stress [39]. However, we found no direct relationship between *Sirt1* and *ACE2* (Figure 2D), possibly due to the species-specific interactions (murine Sirt1 vs. human ACE2)**.** SIRT2 deficiency promotes age-related cardiac hypertrophy and fibrosis [45,47], whereas SIRT3 maintains oxidative metabolism and its loss links to mitochondrial dysfunction and senescence [45,48]. Surprisingly, cardioprotective Sirt6 (acting via adenosine monophosphate-activated protein kinase (AMPK)/ACE2 [49,50]) was upregulated in aged hearts in our study, contrasting with SIRT6-deficient mice progeroid models [51]. This may reflect aging stage specificity.

Telomere attrition, a hallmark of aging, triggers cell cycle arrest and senescence when telomeres reach critical shortening [52]. Shelterin, a protein complex composed of (TRF1, TRF2, TIN2, TPP1, RAP1), protects telomeres; disruption of its subunits induces DNA damage responses and premature aging [53,54,55]. However, the relationship between ACE2 and telomere attrition remains unexplored. Our data revealed severe telomere attrition in aged wild-type hearts, marked by reduced shelterin transcript levels, which was not observed in K18-hACE2 mice (Figure 2E). These findings suggested that ACE2 preserves telomere integrity, thereby delaying cardiac aging.

Given ACE2’s mitigation of aging markers, we investigated its effect on immune senescence, a driver of CVD [56,57,58,59]. Our findings position ACE2 as a modulator of immune homeostasis. Aged hematopoietic stem cells (HSCs) in the circulatory system drive immune senescence via skewed differentiation toward myelopoiesis [38,60,61]. In this study, ACE2 overexpression attenuated age-related increases in LT-HSC (Lin^−^c-kit^+^Sca^−^1^+^CD150^+^CD48^−^) and declines in ST-HSC (Lin^−^c-kit^+^Sca-1^+^CD150^+^CD48^+^) (Figure 3C). Notably, ACE2 rescued CLPs (Lin^−^c-kit^low^Sca-1^low^CD127^+^Flt3^+^) depletion without affecting CMPs (Lin^−^c-kit^+^Sca-1^−^CD34^+^CD16/32^−^), indicating the preferential restoration of lymphopoiesis. Similarly, our study analyzed ACE2 ameliorating lymphopenia in peripheral blood and spleen, which may be explained by the unique ontogeny of these cells, at least in part, due to the weaker reduction in the Lin^−^c-kit^low^Sca-1^low^CD127^+^Flt3^+^ fraction in CLP (Figure 3C). Dendritic cells (DCs) serve as critical bridges between innate and adaptive immunity. Conventional DCs (cDCs) remain stable [61,62,63], whereas plasmacytoid DCs (pDCs) decline with aging [61,64], unaffected by ACE2 overexpression (Supplementary Figure S4A,B). This observation differs from Kovacs’s findings, where *ACE2* knockout mice showed a slight reduction in DCs [65], possibly masked by aging-specific pathologies. We also found neutrophil dynamics were tissue-specific: ACE2 restored peripheral blood (but not splenic) neutrophil (CD11b^+^Ly6g^+^) counts (Supplementary Figure S4A,B), likely attributed to higher soluble ACE2 levels in circulation [15,66]. The trafficking of CD11b^+^Ly6C^int^ and CD11b^+^Ly6C^low^ monocytes, disrupted in aging [67], was preserved in ACE2-overexpressing mice (Figure 3A,B).

Similarly, ACE2 overexpression mitigated age-related peripheral blood macrophage (CD11b^+^F4/80^+^) depletion and M2 (CD11b^+^F4/80^+^CD206^+^) polarization (Figure 3A and Supplementary Figure S4A,B), while splenic macrophage polarization was unaffected, highlighting tissue-specific microenvironment differences. Additionally, the age-dependent expansion of MDSCs (CD11b^+^F4/80^−^), which impair T cell function and promote age-related pathologies [68,69], was observed in aged spleens but not in K18-hACE2 mice (Figure 3A), indicating the role of ACE2 in suppression of MDSCs accumulation.

Aged microenvironments impair NK cell maturation and proliferation [70]. Meanwhile, single-cell RNA sequencing further confirmed age-related declines in NK cell proportions [71]. Consistent with these reports, NK cell (CD3^−^NK1.1^+^) proportions decreased in the peripheral blood of aged wild-type mice; however, this decline was absent in ACE2-overexpressing aged mice (Figure 3A), while this response was not replicated in the spleen (Supplementary Figure S4B), indicating tissue-specific protection. Physiological aging diminishes naive lymphocyte output while expanding memory T cell populations [72]. Consistent with human studies [38], aged mice exhibited reduced naive T (CD3^+^CD4^+^CD44^−^CD62L^+^) and B cells (CD19^+^B220^+^CD93^−^) in peripheral blood—a trend attenuated by ACE2 overexpression that partially restored the naive-to-effector immune cell ratios (Figure 3A,B). Collectively, ACE2 overexpression counteracted HSC differentiation bias and attenuated age-related dysregulation of neutrophils, monocytes, macrophages, and adaptive immune subsets, thereby preserving systemic immune homeostasis.

Given that hACE2-overexpressing mice mimic human COVID-19 pathophysiology with robust preclinical validation, we used this humanized ACE2 model for our experiments. Mouse ACE2 exhibits high sequence homology (Supplementary Figure S6) and conserved catalytic activity with hACE2, supporting the use of hACE2 as a relevant model. Meanwhile, ACE2 is a pivotal component of the RAS. Our group has systematically summarized the mechanisms and molecular basis underlying ACE2’s role in physiological aging and aging-related diseases. The regulation of ACE2 during aging is multifaceted. At the transcriptional level, SIRT1, chitinase 3-like protein 1 (CHI3L1), the Brahma-related gene 1 (Brg1)-Forkhead box protein M1 (FoxM1) complex, and Apelin regulate ACE2 transcription. Post-translational modifications, such as AMPK-mediated phosphorylation and mouse double minute 2 homolog (MDM2)- or S-phase kinase-associated protein 2 (Skp2)-mediated ubiquitination, critically affect ACE2 stability and activity. Catalytically, ACE2 is modulated by its substrate Ang II and specific inhibitors (e.g., competitive inhibitor MLN-4760 and allosteric inhibitor DX600). Moreover, a disintegrin and metalloproteinase 17 (ADAM17) and transmembrane serine protease 2 (TMPRSS2) primarily shed the ACE2 ectodomain to release soluble ACE2 (sACE2), which retains enzymatic activity but loses the signal transduction function associated with tissue anchoring. Collectively, future interventions targeting ACE2 and its related signaling networks may offer new strategies to delay aging and prevent or treat aging-related diseases.

## 4. Materials and Methods

### 4.1. Animals

Female C57BL/6 mice at 8 weeks (young) and 48 weeks (aged) of age were purchased from Beijing Vital River Laboratory Animal Technology Co., Ltd. (Beijing, China). Age-matched transgenic K18-hACE2 female mice at 10–12 weeks (young) and 42–50 weeks (aged) were obtained from Jiangsu GemPharmatech Co., Ltd. (Nanjing, China). All mice were maintained under specific pathogen-free (SPF) conditions at controlled ambient temperature (22 ± 1 °C) and relative humidity (50 ± 10%) with ad libitum access to standard chow and sterile water. All experimental procedures were approved by the Animal Research Committee of Tianjin University of Traditional Chinese Medicine (Ethics Approval No. TCM-LAEC2023228c1534; Approval Date: 4 October 2023) in compliance with the NIH Guide for the Care and Use of Laboratory Animals. An ACE2-humanized mouse model was generated on the C57BL/6JGpt genetic background using gene-editing technology (Supplementary Figure S5). Specifically, human cytokeratin 18 (K18) promoter-driven overexpression of hACE2 was targeted to the safe harbor H11 locus, resulting in the establishment of a K18-hACE2 overexpression mouse strain [73]. Hemizygous transgenic animals (6–8 weeks old) were bred with wildtype C57BL/6 male mice (8–10 weeks old) to generate experimental groups. Genomic integration of the transgene in the offspring was analyzed by PCR on DNA extracted from tail biopsies. The following primer sets were used: a set specific for the 5′ arm integration site (5′arm-F: 5′-GGGCAGTCTGGTACTTCCAAGCT-3′ and 5′arm-R: 5′-CATACAAGGCTTCTGGGAGGTAAG-3′) and a set specific for the wild-type allele (WT-F: 5′-CAGCAAAACCTGGCTGTGGATC-3′ and WT-R: 5′-ATGAGCCACCATGTGGGTGTC-3′). Adult transgenic animals and wild type females at different ages were used in the experiments.

### 4.2. Organ Index and Morphology

Following retro-orbital blood collection, mice were euthanized via cervical dislocation under anesthesia. The heart, liver, spleen, pairs of lungs and kidneys were surgically excised, weighed, and normalized to body weight. For histopathological analysis, cardiac tissues (transversely sectioned at the midventricular level, with the apical portion reserved for fixation) were fixed in 4% paraformaldehyde (P1110, Solarbio Life Sciences, Beijing, China) and dehydrated in 25% sucrose, embedded in Tissue-Tek optimal cutting temperature compound (4583, Sakura Finetek, Torrance, CA, USA), and cryosectioned at 5 μm thickness. Sections were stained with hematoxylin and eosin (G1120, Solarbio Life Sciences, Beijing, China) for morphological evaluation.

### 4.3. Isolation of Hematopoietic Stem Cells

Femurs and tibiae were dissected, and bone marrow was flushed with ice-cold phosphate-buffered saline (PBS). The cell suspension was filtered through a 70 μm nylon mesh strainer (BS-70-CS, Biosharp Life Sciences, Hefei, China), centrifuged at 1000 rpm for 8 min at 4 °C, and treated with red blood cell lysis buffer (R1010, Solarbio Life Sciences, Beijing, China) for erythrocyte depletion. After washing, the hematopoietic stem cell (HSC)-enriched pellet was resuspended and filtered for flow cytometry experiments (BD FACSymphony™ A1, BD Bioscience, Franklin Lakes, NJ, USA).

### 4.4. Quantitative Reverse-Transcription Polymerase Chain Reaction

Total RNA was extracted from cardiac tissue adjacent to the atrial appendage using TRIzol reagent (15596018, Thermo Fisher Scientific, Waltham, MA, USA) following the manufacturer’s protocol. Complementary deoxyribonucleic acid (cDNA) was synthesized using a Hifair^®^ III First-Strand cDNA Synthesis Kit (11139ES60, Yeasen Biotechnology, Shanghai, China), and quantitative PCR (qPCR) was performed using the SYBR Green method with the quantitative PCR system (Eppendorf, Hamburg, Germany). The mRNA expression was normalized to *Gapdh* and calculated via the 2^−ΔΔCt^ method. All primer sequences (Sangon Biotech, Shanghai, China) are detailed in Supplementary Table S1.

### 4.5. Flow Cytometry

Spleens and peripheral blood samples were mechanically dissociated and sequentially filtered through 70 μm cell strainers to generate single-cell suspensions. Erythrocytes were lysed using RBC lysing buffer. Cells were pre-blocked with anti-mouse CD16/CD32 monoclonal antibody (156614, BioLegend, San Diego, CA, USA) for 15 min at 4 °C, followed by staining with fluorescein-conjugated antibodies for 30 min at 4 °C in the dark. Flow cytometry data were acquired on a BD FACSymphony™ A1 cytometer (BD Bioscience, Franklin Lakes, NJ, USA) and analyzed using FlowJo.10.8.1 software. All antibodies are listed in Supplementary Table S2. The gating strategy is illustrated in Supplementary Figures S1–S3.

### 4.6. Statistical Analysis

All data are presented as the mean ± standard error of mean (SEM). Statistical analyses were performed using GraphPad Prism 8.0. Comparisons between two groups were analyzed using unpaired Student’s *t*-test. Differences among groups were analyzed using one-way ANOVA for multiple comparisons. Statistical significance was defined as *p* < 0.05.

## 5. Conclusions

**Figure 4:**
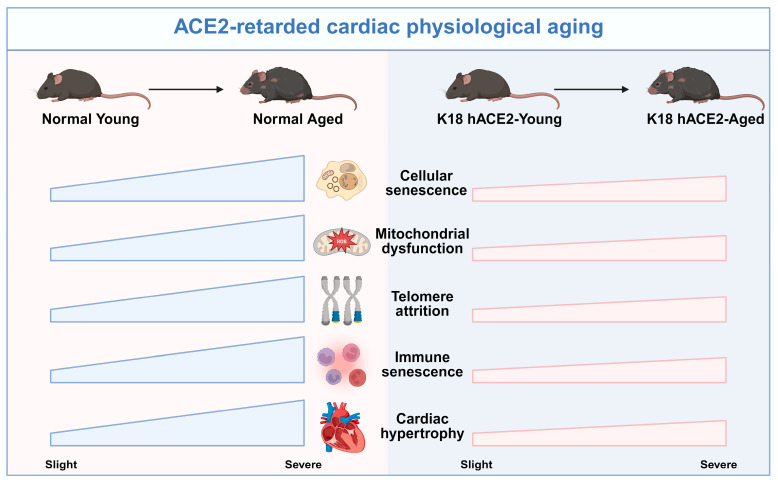
ACE2-retarded cardiac physiological aging. Created in BioRender. –H, f%. (2026) https://BioRender.com/ng9gklq.

In conclusion, our study demonstrated that systemic ACE2 overexpression ameliorated cardiac degeneration during early physiological aging in C57BL/6 mice, and the underlying mechanisms involved the amelioration of mitochondrial dysfunction, telomere attrition and immune system dysregulation (Figure 4).

## 6. Restriction

Due to batch limitations, our original qRT-PCR and flow cytometry analyses were performed with samples grouped by genotype, preventing direct cross-genotype normalization. To address this issue, we subsequently re-analyzed a subset of key samples with all four groups processed together and normalized to young WT. Preliminary qRT-PCR and flow cytometry data suggest that ACE2 overexpression delays aging in mice. Further studies using Western blotting and other methods are required to clarify the underlying mechanisms. Nevertheless, the exclusive use of female animals in this study limits the generalizability of the conclusions. Future studies should include male mice to determine whether the observed anti-aging effect of ACE2 is sex-dependent or conserved across sexes. The K18 promoter used in this model does not confer cardiomyocyte-specific ACE2 overexpression. Thus, future studies employing a heart-specific promoter (e.g., Myh6/α-MHC) are necessary to confirm the cell-autonomous role of ACE2 in cardiac aging.

## Data Availability

The corresponding authors will provide the datasets supporting this study upon reasonable request.

## Supplementary Materials

The following supporting information can be downloaded at: https://www.mdpi.com/article/10.3390/ijms27115082/s1.

## References

[1] Makino K., Lee S., Bae S., Chiba I., Harada K., Katayama O., Shinkai Y., Shimada H. (2021). Absolute Cardiovascular Disease Risk Assessed in Old Age Predicts Disability and Mortality: A Retrospective Cohort Study of Community-Dwelling Older Adults. J. Am. Heart Assoc..

[2] Luan Y., Zhu X.F., Jiao Y.X., Liu H., Huang Z., Pei J.Y., Xu Y.W., Yang Y., Ren K.D. (2024). Cardiac cell senescence: Molecular mechanisms, key proteins and therapeutic targets. Cell Death Discov..

[3] Lakatta E.G., Levy D. (2003). Arterial and Cardiac Aging: Major Shareholders in Cardiovascular Disease Enterprises. Circulation.

[4] Lakatta E.G. (1990). Changes in cardiovascular function with aging. Eur. Heart J..

[5] Buford T.W. (2016). Hypertension and aging. Ageing Res. Rev..

[6] Wang J.C., Bennett M. (2012). Aging and Atherosclerosis. Circ. Res..

[7] Li H., Hastings M.H., Rhee J., Trager L.E., Roh J.D., Rosenzweig A. (2020). Targeting Age-Related Pathways in Heart Failure. Circ. Res..

[8] Tucker N.R., Chaffin M., Bedi K.C., Papangeli I., Akkad A.D., Arduini A., Hayat S., Eraslan G., Muus C., Bhattacharyya R.P. (2020). Myocyte Specific Upregulation of ACE2 in Cardiovascular Disease: Implications for SARS-CoV-2 Mediated Myocarditis. Circulation.

[9] Nicin L., Abplanalp W.T., Mellentin H., Kattih B., Tombor L., John D., Schmitto J.D., Heineke J., Emrich F., Arsalan M. (2020). Cell type-specific expression of the putative SARS-CoV-2 receptor ACE2 in human hearts. Eur. Heart J..

[10] Patel V.B., Zhong J.C., Fan D., Basu R., Morton J.S., Parajuli N., McMurtry M.S., Davidge S.T., Kassiri Z., Oudit G.Y. (2014). Angiotensin-converting enzyme 2 is a critical determinant of angiotensin II-induced loss of vascular smooth muscle cells and adverse vascular remodeling. Hypertension.

[11] Gallagher P.E., Ferrario C.M., Tallant E.A. (2008). Regulation of ACE2 in cardiac myocytes and fibroblasts. Am. J. Physiol.-Heart Circ. Physiol..

[12] Donoghue M., Hsieh F., Baronas E., Godbout K., Gosselin M., Stagliano N., Donovan M., Woolf B., Robison K., Jeyaseelan R. (2000). A novel angiotensin-converting enzyme-related carboxypeptidase (ACE2) converts angiotensin I to angiotensin 1-9. Circ. Res..

[13] Tipnis S.R., Hooper N.M., Hyde R., Karran E., Christie G., Turner A.J. (2000). A Human Homolog of Angiotensin-converting Enzyme. J. Biol. Chem..

[14] Patel V.B., Clarke N., Wang Z., Fan D., Parajuli N., Basu R., Putko B., Kassiri Z., Turner A.J., Oudit G.Y. (2014). Angiotensin II induced proteolytic cleavage of myocardial ACE2 is mediated by TACE/ADAM-17: A positive feedback mechanism in the RAS. J. Mol. Cell. Cardiol..

[15] Chen Q., Li Y., Bie B., Zhao B., Zhang Y., Fang S., Li S., Zhang Y. (2023). P38 MAPK activated ADAM17 mediates ACE2 shedding and promotes cardiac remodeling and heart failure after myocardial infarction. Cell Commun. Signal..

[16] Zhong J., Basu R., Guo D., Chow F.L., Byrns S., Schuster M., Loibner H., Wang X.H., Penninger J.M., Kassiri Z. (2010). Angiotensin-Converting Enzyme 2 Suppresses Pathological Hypertrophy, Myocardial Fibrosis, and Cardiac Dysfunction. Circulation.

[17] Oudit G.Y., Kassiri Z., Patel M.P., Chappell M., Butany J., Backx P.H., Tsushima R.G., Scholey J.W., Khokha R., Penninger J.M. (2007). Angiotensin II-mediated oxidative stress and inflammation mediate the age-dependent cardiomyopathy in ACE2 null mice. Cardiovasc. Res..

[18] Patel V.B., Bodiga S., Fan D., Das S.K., Wang Z., Wang W., Basu R., Zhong J., Kassiri Z., Oudit G.Y. (2012). Cardioprotective Effects Mediated by Angiotensin II Type 1 Receptor Blockade and Enhancing Angiotensin 1-7 in Experimental Heart Failure in Angiotensin-Converting Enzyme 2–Null Mice. Hypertension.

[19] Ye M., Wysocki J., Gonzalez-Pacheco F.R., Salem M., Evora K., Garcia-Halpin L., Poglitsch M., Schuster M., Batlle D. (2012). Murine Recombinant Angiotensin-Converting Enzyme 2: Effect on Angiotensin II Dependent Hypertension and Distinctive ACE2 Inhibitor Characteristics on rodent and human ACE2. Hypertension.

[20] Sanad A.M., Qadri F., Popova E., Rodrigues A.F., Heinbokel T., Quach S., Schulz A., Bachmann S., Kreutz R., Alenina N. (2023). Transgenic angiotensin-converting enzyme 2 overexpression in the rat vasculature protects kidneys from ageing-induced injury. Kidney Int..

[21] López-Otín C., Blasco M.A., Partridge L., Serrano M., Kroemer G. (2013). The Hallmarks of Aging. Cell.

[22] López-Otín C., Blasco M.A., Partridge L., Serrano M., Kroemer G. (2023). Hallmarks of aging: An expanding universe. Cell.

[23] Abdellatif M., Rainer P.P., Sedej S., Kroemer G. (2023). Hallmarks of cardiovascular ageing. Nat. Rev. Cardiol..

[24] Silva R.A.P., Chu Y., Miller J.D., Mitchell I.J., Penninger J.M., Faraci F.M., Heistad D.D. (2012). Impact of ACE2 Deficiency and Oxidative Stress on Cerebrovascular Function With Aging. Stroke.

[25] Goetze J.P., Bruneau B.G., Ramos H.R., Ogawa T., de Bold M.K., de Bold A.J. (2020). Cardiac natriuretic peptides. Nat. Rev. Cardiol..

[26] Zhang W., Che Y., Tang X., Chen S., Song M., Wang L., Sun A., Chen H., Xu M., Wang M. (2023). A biomarker framework for cardiac aging: The Aging Biomarker Consortium consensus statement. Life Med..

[27] Yamamoto K., Ohishi M., Katsuya T., Ito N., Ikushima M., Kaibe M., Tatara Y., Shiota A., Sugano S., Takeda S. (2006). Deletion of Angiotensin-Converting Enzyme 2 Accelerates Pressure Overload-Induced Cardiac Dysfunction by Increasing Local Angiotensin II. Hypertension.

[28] Crackower M.A., Sarao R., Oudit G.Y., Yagil C., Kozieradzki I., Scanga S.E., Oliveira dos Santos A.J., da Costa J., Zhang L., Pei Y. (2002). Angiotensin-converting enzyme 2 is an essential regulator of heart function. Nature.

[29] Gurley S.B., Allred A., Le T.H., Griffiths R., Mao L., Philip N., Haystead T.A., Donoghue M., Breitbart R.E., Acton S.L. (2006). Altered blood pressure responses and normal cardiac phenotype in ACE2-null mice. J. Clin. Investig..

[30] Matsushita H., Chang E., Glassford A.J., Cooke J.P., Chiu C.P., Tsao P.S. (2001). eNOS Activity Is Reduced in Senescent Human Endothelial Cells. Circ. Res..

[31] Sampaio W.O., Souza dos Santos R.A., Faria-Silva R., da Mata Machado L.T., Schiffrin E.L., Touyz R.M. (2007). Angiotensin-(1-7) Through Receptor Mas Mediates Endothelial Nitric Oxide Synthase Activation via Akt-Dependent Pathways. Hypertension.

[32] Epelman S., Tang W., Chen S., Van L.F., Francis G., Sen S. (2008). Detection of Soluble Angiotensin-Converting Enzyme 2 in Heart Failure. J. Am. Coll. Cardiol..

[33] Mao Z., Ke Z., Gorbunova V., Seluanov A. (2012). Replicatively senescent cells are arrested in G1 and G2 phases. Aging.

[34] Chen M.S., Lee R.T., Garbern J.C. (2022). Senescence mechanisms and targets in the heart. Cardiovasc. Res..

[35] Rufini A., Tucci P., Celardo I., Melino G. (2013). Senescence and aging: The critical roles of p53. Oncogene.

[36] Takeshita H., Yamamoto K., Nozato S., Takeda M., Fukada S.i., Inagaki T., Tsuchimochi H., Shirai M., Nozato Y., Fujimoto T. (2018). Angiotensin-converting enzyme 2 deficiency accelerates and angiotensin 1-7 restores age-related muscle weakness in mice. J. Cachexia Sarcopenia Muscle.

[37] Takeshita H., Yamamoto K., Mogi M., Nozato S., Rakugi H. (2023). Is the anti-aging effect of ACE2 due to its role in the renin-angiotensin system?—Findings from a comparison of the aging phenotypes of ACE2-deficient, Tsukuba hypertensive, and Mas-deficient mice. Hypertens. Res..

[38] Li X., Li C., Zhang W., Wang Y., Qian P., Huang H. (2023). Inflammation and aging: Signaling pathways and intervention therapies. Signal Transduct. Target. Ther..

[39] Clarke N.E., Belyaev N.D., Lambert D.W., Turner A.J. (2013). Epigenetic regulation of angiotensin-converting enzyme 2 (ACE2) by SIRT1 under conditions of cell energy stress. Clin. Sci..

[40] Valenzuela R., Rodriguez Perez A.I., Costa Besada M.A., Rivas Santisteban R., Garrido Gil P., Lopez Lopez A., Navarro G., Lanciego J.L., Franco R., Labandeira Garcia J.L. (2021). An ACE2/Mas-related receptor MrgE axis in dopaminergic neuron mitochondria. Redox Biol..

[41] Moran C.S., Biros E., Krishna S.M., Wang Y., Tikellis C., Morton S.K., Moxon J.V., Cooper M.E., Norman P.E., Burrell L.M. (2017). Resveratrol Inhibits Growth of Experimental Abdominal Aortic Aneurysm Associated With Upregulation of Angiotensin-Converting Enzyme 2. Arterioscler. Thromb. Vasc. Biol..

[42] Wan T.T., Li Y., Li J.X., Xiao X., Liu L., Li H.H., Guo S.B. (2024). ACE2 activation alleviates sepsis-induced cardiomyopathy by promoting MasR-Sirt1-mediated mitochondrial biogenesis. Arch. Biochem. Biophys..

[43] Shi T.T., Yang F.Y., Liu C., Cao X., Lu J., Zhang X.L., Yuan M.X., Chen C., Yang J.K. (2018). Angiotensin-converting enzyme 2 regulates mitochondrial function in pancreatic β-cells. Biochem. Biophys. Res. Commun..

[44] Bi S.J., Jiang X.Y., Ji Q.Z., Wang Z.H., Ren J., Wang S., Yu Y., Wang R.Q., Liu Z.P., Liu J.H. (2024). The sirtuin-associated human senescence program converges on the activation of placenta-specific gene PAPPA. Dev. Cell.

[45] Pukhalskaia A.E., Kvetnoy I.M., Linkova N.S., Diatlova A.S., Gutop E.O., Kozlov K.L., Paltsev M.A. (2023). Sirtuins and Aging. Neurosci. Behav. Physiol..

[46] Alcendor R.R., Gao S., Zhai P., Zablocki D., Holle E., Yu X., Tian B., Wagner T., Vatner S.F., Sadoshima J. (2007). Sirt1 Regulates Aging and Resistance to Oxidative Stress in the Heart. Circ. Res..

[47] Ye Y.X., Yang K., Liu H.S., Yu Y., Song M.S., Huang D.Y., Lei J.H., Zhang Y.Y., Liu Z.P., Chu Q. (2023). SIRT2 counteracts primate cardiac aging via deacetylation of STAT3 that silences CDKN2B. Nat. Aging.

[48] Diao Z., Ji Q., Wu Z., Zhang W., Cai Y., Wang Z., Hu J., Liu Z., Wang Q., Bi S. (2021). SIRT3 consolidates heterochromatin and counteracts senescence. Nucleic Acids Res..

[49] Abdel-Nasser Z.M., Zaafan M.A., Abdelhamid A.M. (2023). Modulation of the miR-122/Sirt-6/ACE2 axis on experimentally-induced myocardial infarction. Chem.-Biol. Interact..

[50] Song J.J., Yang M., Liu Y., Song J.W., Wang J., Chi H.J., Liu X.Y., Zuo K., Yang X.C., Zhong J.C. (2020). MicroRNA-122 aggravates angiotensin II-mediated apoptosis and autophagy imbalance in rat aortic adventitial fibroblasts via the modulation of SIRT6-elabela-ACE2 signaling. Eur. J. Pharmacol..

[51] Mostoslavsky R., Chua K.F., Lombard D.B., Pang W.W., Fischer M.R., Gellon L., Liu P., Mostoslavsky G., Franco S., Murphy M.M. (2006). Genomic instability and aging-like phenotype in the absence of mammalian SIRT6. Cell.

[52] Mir S.M., Samavarchi Tehrani S., Goodarzi G., Jamalpoor Z., Jahanbakhsh A., Khelghati N., Qujeq D., Maniati M. (2020). Shelterin Complex at Telomeres: Implications in Ageing. Clin. Interv. Aging.

[53] van Steensel B., Smogorzewska A., de Lange T. (1998). TRF2 protects human telomeres from end-to-end fusions. Cell.

[54] Chakravarti D., LaBella K.A., DePinho R.A. (2021). Telomeres: History, health, and hallmarks of aging. Cell.

[55] Yang Z., Takai K.K., Lovejoy C.A., de Lange T. (2020). Break-induced replication promotes fragile telomere formation. Genes Dev..

[56] Del Pinto R., Ferri C. (2018). Inflammation-Accelerated Senescence and the Cardiovascular System: Mechanisms and Perspectives. Int. J. Mol. Sci..

[57] Swirski F.K., Nahrendorf M. (2018). Cardioimmunology: The immune system in cardiac homeostasis and disease. Nat. Rev. Immunol..

[58] Rurik J.G., Tombácz I., Yadegari A., Méndez Fernández P.O., Shewale S.V., Li L., Kimura T., Soliman O.Y., Papp T.E., Tam Y.K. (2022). CAR T cells produced in vivo to treat cardiac injury. Science.

[59] Amor C., Feucht J., Leibold J., Ho Y.J., Zhu C.Y., Alonso Curbelo D., Mansilla Soto J., Boyer J.A., Li X., Giavridis T. (2020). Senolytic CAR T cells reverse senescence-associated pathologies. Nature.

[60] Rundberg Nilsson A., Soneji S., Adolfsson S., Bryder D., Pronk C.J. (2016). Human and Murine Hematopoietic Stem Cell Aging Is Associated with Functional Impairments and Intrinsic Megakaryocytic/Erythroid Bias. PLoS ONE.

[61] Krishnarajah S., Ingelfinger F., Friebel E., Cansever D., Amorim A., Andreadou M., Bamert D., Litscher G., Lutz M., Mayoux M. (2021). Single-cell profiling of immune system alterations in lymphoid, barrier and solid tissues in aged mice. Nat. Aging.

[62] Jiang R.D., Liu M.Q., Chen Y., Shan C., Zhou Y.W., Shen X.R., Li Q., Zhang L., Zhu Y., Si H.R. (2020). Pathogenesis of SARS-CoV-2 in Transgenic Mice Expressing Human Angiotensin-Converting Enzyme 2. Cell.

[63] Chen A., Jaiswal S., Martinez D., Yerinde C., Ji K., Miranda V., Fung M.E., Weiss S.A., Zschummel M., Taguchi K. (2024). The aged tumor microenvironment limits T cell control of cancer. Nat. Immunol..

[64] Splunter M.V., Perdijk O., Fick Brinkhof H., Floris Vollenbroek E.G., Meijer B., Brugman S., Savelkoul H., Van Hoffen E., Joost van Neerven R.J. (2019). Plasmacytoid dendritic cell and myeloid dendritic cell function in ageing: A comparison between elderly and young adult women. PLoS ONE.

[65] Kovacs A., Ipsen A., Manzel A., Linker R.A. (2013). ACE2 drives dendritic cell function and neuroantigen specific immune responses. Brain Behav. Immun..

[66] Wang C.W., Chuang H.C., Tan T.H. (2023). ACE2 in chronic disease and COVID-19: Gene regulation and post-translational modification. J. Biomed. Sci..

[67] Blacher E., Tsai C., Litichevskiy L., Shipony Z., Iweka C.A., Schneider K.M., Chuluun B., Heller H.C., Menon V., Thaiss C.A. (2022). Aging disrupts circadian gene regulation and function in macrophages. Nat. Immunol..

[68] Lee K.A., Flores R.R., Jang I.H., Saathoff A., Robbins P.D. (2022). Immune Senescence, Immunosenescence and Aging. Front. Aging.

[69] Flores R.R., Clauson C.L., Cho J., Lee B.C., McGowan S.J., Baker D.J., Niedernhofer L.J., Robbins P.D. (2017). Expansion of myeloid-derived suppressor cells with aging in the bone marrow of mice through a NF-κB-dependent mechanism. Aging Cell.

[70] Brauning A., Rae M., Zhu G., Fulton E., Admasu T.D., Stolzing A., Sharma A. (2022). Aging of the Immune System: Focus on Natural Killer Cells Phenotype and Functions. Cells.

[71] Mogilenko D.A., Shpynov O., Andhey P.S., Arthur L., Swain A., Esaulova E., Brioschi S., Shchukina I., Kerndl M., Bambouskova M. (2021). Comprehensive Profiling of an Aging Immune System Reveals Clonal GZMK+ CD8+ T Cells as Conserved Hallmark of Inflammaging. Immunity.

[72] Caruso C., Ligotti M.E., Accardi G., Aiello A., Candore G. (2022). An immunologist’s guide to immunosenescence and its treatment. Expert. Rev. Clin. Immunol..

[73] Winkler E.S., Bailey A.L., Kafai N.M., Nair S., McCune B.T., Yu J., Fox J.M., Chen R.E., Earnest J.T., Keeler S.P. (2020). SARS-CoV-2 infection of human ACE2-transgenic mice causes severe lung inflammation and impaired function. Nat. Immunol..

