# Supplementary material for: Angiotensin-Converting Enzyme 2 Overexpression Protects Heart from Aging-Induced Injury in C57BL/6 Mice
Source: Int J Mol Sci. 2026 Jun 4;27(11):5082. doi: 10.3390/ijms27115082 (PMC13257251; doi:10.3390/ijms27115082)
Supplement: Supplementary file 1 [file ijms-27-05082-s001.zip › ijms-4276341-supplementary.pdf]

Supplementary Table S1. Primer used for RT-qPCR.

Target gene	Forword primer sequence	Reserve primer sequence
ACE2	TCCACGATCCCATGCCTATG	TGCTGAAGACCCACTTTGCT
ANP	ATTGACAGGATTGGAGCCCAGAGT	TGACACACCACAAGGGCTTAGGAT
Atg5	TGCGGTTGAGGCTCACTTTATGTC	GTCCCATCCAGAGCTGCTTGTG
Atg7	GGCACGAACTGACCCAGAAGAAG	GCAGACCAGCAGAGTCACCATTG
Atg9b	ATCAACAGCAGCAGCAAGAACTATG	GAAGCAGGACTGGAGCCATCAC
Bax	CGTGGTTGCCCTCTTCTACTTTG	TCCAGTGTCCAGCCCATGATG
Bcl-2	TACGAGTGGGATGCTGGAGATG	TCAGGCTGGAAGGAGAAGATGC
Becn1	AGGCAGTGGCGGCTCCTATTC	TGAGGACACCCAGGCAAGACC
BNP	TAACGCACTGAAGTTGTTGTAGG	CGCTATGTTTATTATGTTGTGGC
Cat	CCATAGCCAGAAGAGAAACCCACAG	GGAATCCCTCGGTCACTGAACAAG
COX I	TCAACCTTGTCAACACAGCCTCAC	GGCACACGGAAGGAAACATAGGG
cTnt	AGGAGGCAGTGGAGGAGGAG	CTGGACCTTCTTCAGCATCTTTGG
CXCL-10	GCCTCATCCTGCTGGGTCTG	TTCCCTATGGCCCTCATTCTCAC
eNOS	AGGCATCACCAGGAAGAAGACC	CCTTCACACGCTTCGCCATC
ERK	TGAAGACACAGCACCTCAGCAATG	GGTGTTCAGCAGGAGGTTGGAAG
FOXO-3α	CCGTGAGCAAGCCGTGTACTG	TATCCAGCAGGTCGTCCATGAGG
HO-1	AAGACCGCCTTCCTGCTCAAC	TCTGACGAAGTGACGCCATCTG
HP1	AGATTCCTGCGGTGACTTAATGTTC	AATCTGTGGACACTTCACGTTAGC
IFN-γ	CGCTACACACTGCATCTTGG	TTCCACATCTATGCCACTTGAG
IL-10	GGACAACATACTGCTAACCGACTC	TGGATCATTTCCGATAAGGCTTGG
IL-1β	CTCGCAGCAGCACATCAACAAG	CCACGGGAAAGACACAGGTAGC
IL-6	TTCTTGGGACTGATGCTGGT	CAGGTCTGTTGGGAGTGGTA
IL-7	AATCGTGCTGCTCGCAAGTTG	ACCAGTGTTTGTGTGCCTTGTG
JNK	CGCCTTATGTGGTGACTCGCTAC	CTCCCATGATGCACCCAACTGAC
LC3B	AGCCTTCTTCCTCCTGGTGAATG	TGCTGTCCCGAATGTCTCCTG
MCP-1	CTTCTGGGCCTGCTGTTCA	CCAGCCTACTCATTGGGATCA
Mmp3	GACGATGATGAACGATGGACAGAG	CCTTGGCTGAGTGGTAGAGTCC
mTOR	TCCGACCGTCCGCCTTCAC	GCAGTCCGTTCCTTCTCCTTCTTG
NQO1	TCCTGCGTTTCTGTGGCTTCC	TCCAGACGTTTCTTCCATCCTTCC
Nrf1	GTGAATTACTCTGCTGTGGCTGATG	TGCTTGCGTCGTCTGGATGGT
Nrf2	ACTACAGTCCCAGCAGAGTGATGG	GCGTGCTCAGAAACCTCCTTCC
p16	TCAAGACATCGTGCGATATTTG	TTAGCTCTGCTCTTGGGATTG
p21	ATGTCCAATCCTGGTGATGTC	GAAGTCAAAGTTCCACCGTTC
p38	CTGGCTCGGCACACTGATGATG	GCCCACGGACCAAATATCCACTG
p53	TGGAAGGAAATTTGTATCCCGA	GTGGATGGTGGTATACTCAGAG
p62	AGCACAGGCACAGAAGACAAGAG	TCCCACCGACTCCAAGGCTATC
Parkin	GCTTGACACGAGTGGACCTGAG	GGACCTCTGGCTGCTTCTGAATC
PGC1α	CGATGACCCTCCTCACACCAAAC	TTGCGACTGCGGTTGTGTATGG
POT-1α	AGGAGGAAGGCTCTGCGAAGTC	ACGGGGCTGAAAGGGTAAGGAG
RAP1	AGTCGCTCAAGGACCGCTACC	TTCTGTGGCTCTCCGCTATCCG
RB	AGCCTCAGCCTTCCATACTCAG	TCGGAGATATGCTAGACGGTACAC

α-SMA	GCGGGCATCCACGAAAC	TTGATCTTCATGGTGCTGGGT
Rela	GTATTGCTGTGCCTACCCGAAAC	GCTGAGGGATGCTGGGAAGG
Sirt1	CCAGACCTCCCAGACCCTCAAG	GTGACACAGAGACGGCTGGAAC
Sirt2	CCAACCATCTGCCACTACTTCATC	CTCGTTCCAGCGTGTCTATGTTC
Sirt3	GCTGCTTCTGCGGCTCTATACAC	CAAAGGTCCCGTGGGCTTCAAC
Sirt6	GGATGGACCCTGCGTGCTAG	GGCTGTTGGGCTTGGACTTATAC
STAT3	GCGGAGAAGCATTGTGAGTGAG	AGACGGTCCAGGCAGATGTTG
Tfam	TCGCATCCCCTCGTCTATCAGTC	TGGGTAGCTGTTCTGTGGAAAATCG
TIN2	AGCCAGTCTGCACGGAGGAG	AGGCGGTGATGAGTGTCTTAGAGG
TPP1	GAAGACACAAGTTCCGCCCTAGC	AACATTGCTGAAGCCTCCACCAC
Trf1	GAGCAGGAGGAGGTGGGAGAC	GCACAGAGACAGGCAGAGGAAG
Trf2	CATCACCAGCCCACAAACACAAG	TGTCCTCCTCCAACAGCAATCTG
UCP2	CAGATGTGGTAAAGGTCCGCTTCC	TCGTGCAATGGTCTTGTAGGCTTC
α-MHC	CCAGAAGCCTCGCAATGTCAAG	TTTCCAGCCAGCCCATAATGTTG
β-MHC	ACGGCACTGAAGAGGCTGAC	GTATGACACCTGCTGGACATTCTG
Gapdh	CATCACTGCCACCCAGAAGACTG	ATGCCAGTGAGCTTCCCGTTCAG

Supplementary Table S2. Antibodies used for Flow Cytometry

Antibody	Corporation	Product no	Antibody name
CD3	BD Pharmingen	557596	APC-Cy7 Hamster Anti-Mouse CD3e (145-2C11)
CD4	BD Pharmingen	553051	APC Rat Anti-Mouse CD4 (RM4-5)
CD44	BD Pharmingen	563971	BV711 Rat Anti-Mouse CD44 (IM7)
CD62L	BD Pharmingen	564108	BV650 Rat Anti-Mouse CD62L (MEL-14)
CD25	BD Pharmingen	562606	BV421 Rat Anti-Mouse CD25 (PC61)
FOXP3	BD Pharmingen	563101	PE Rat anti-Mouse Foxp3 (R16-715)
CD19	BD Pharmingen	562956	BV510 Rat Anti-Mouse CD19 (1D3)
B220	BD Pharmingen	553087	FITC Rat Anti-Mouse CD45R/B220 (RA3-6B2)
CD93	BD Pharmingen	740941	BV786 Rat Anti-Mouse CD93 (AA4.1)
CD38	BD Pharmingen	740361	BV605 Rat Anti-Mouse CD38 (90/CD38)
IgM	BD Pharmingen	552867	PE-Cy7 Rat Anti-Mouse IgM (R6-60.2)
NK1.1	Biolegend	108728	PerCP/Cyanine5.5 anti-mouse NK-1.1
CD11b	BD Pharmingen	562950	BV510 Rat Anti-CD11b (M1/70)
Ly6c	BD Pharmingen	560596	APC-Cy7 Rat Anti-Mouse Ly-6C (AL-21)
Ly6g	BD Pharmingen	560602	PerCP-Cy5.5 Rat Anti-Mouse Ly-6G (1A8)
CD11c	BD Pharmingen	557401	PE Hamster Anti-Mouse CD11c (HL3)
F4/80	BD Pharmingen	565411	BV421 Rat Anti-Mouse F4/80 (T45-2342)
CD86	BD Pharmingen	560582	PE-Cy7 Rat Anti-Mouse CD86 (GL1)
CD206	Biolegend	141708	APC anti-mouse CD206 (MMR) Antibody
I-A/I-E	BD Pharmingen	563413	BV605 Rat Anti-Mouse I-A/I-E (M5/114.15.2)
CD8	BD Pharmingen	551162	PerCP-Cy5.5 Rat Anti-Mouse CD8a (53-6.7)
LIN	BD Pharmingen	561317	PerCP-Cy5.5 Mouse Lineage Antibody Cocktail
c-kit	BD Pharmingen	558163	PE-Cy7 Rat anti-Mouse CD117 (2B8)
Sca-1	BD Pharmingen	565507	BV510 Rat Anti-Mouse Ly-6A/E (D7)
CD150	Biolegend	115941	Brilliant Violet 711™ anti-mouse CD150 (SLAM)
CD48	Biolegend	103441	Brilliant Violet 605™ anti-mouse CD48
CD127	BD Pharmingen	564175	APC Rat Anti-Mouse CD127 (SB/199)
Flt3	BD Pharmingen	553842	PE Rat Anti-Mouse CD135 (A2F10.1)
CD16/32	Biolegend	156614	FITC anti-mouse CD16/32 Antibody
CD34	BD Pharmingen	562608	BV421 Rat Anti-Mouse CD34 (RAM34)

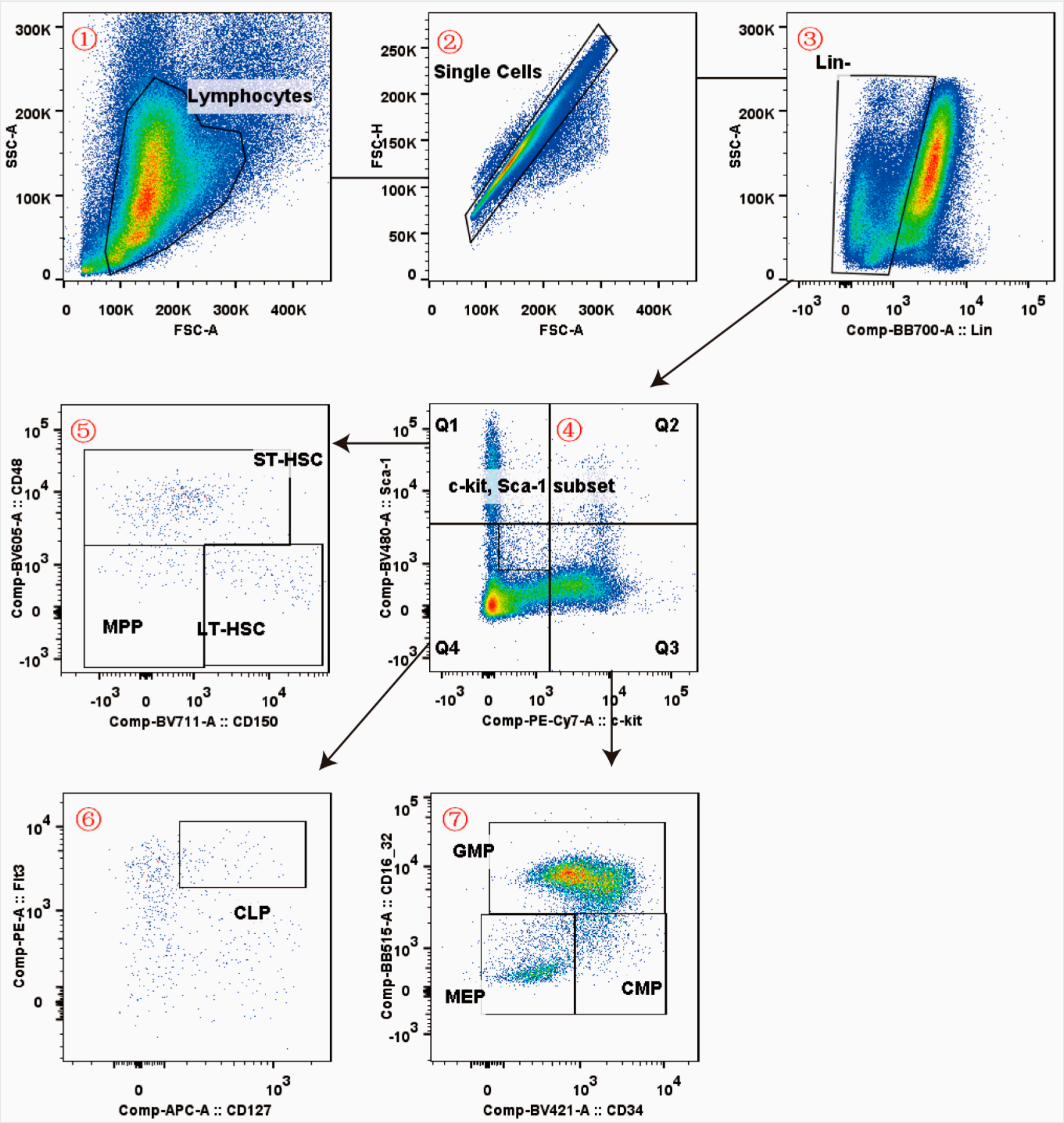

Supplementary Figure S1. Gating strategies for the LT-HSC, ST-HSC, MPP, GMP, CLP, and CMP panel.

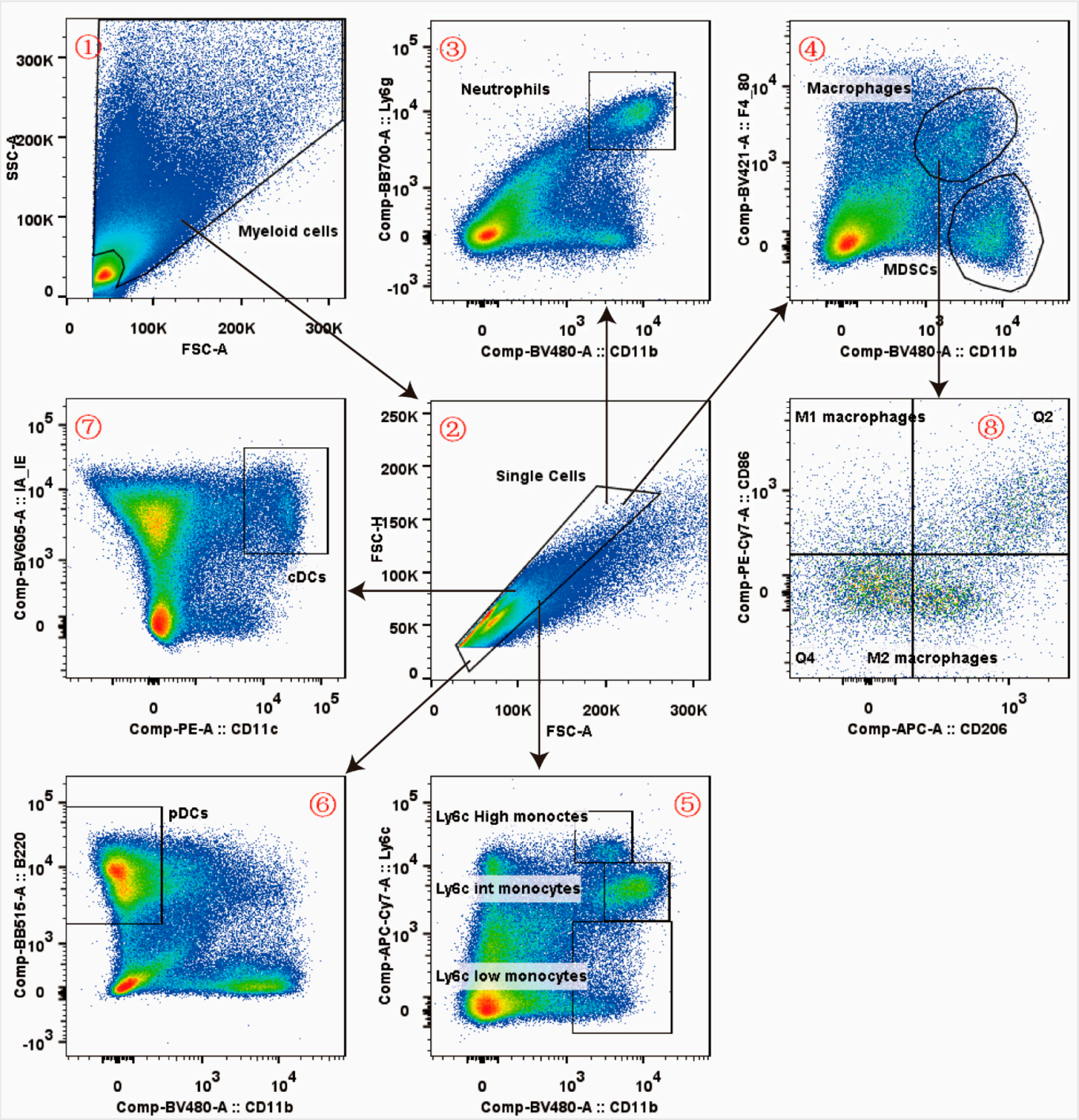

Supplementary Figure S2. Gating strategies for the myeloid cells, neutrophils, macrophages, M1 macrophages, M2 macrophages, MDSCs, Ly6c^{hi} monocytes, Ly6c^{int} monocytes, Ly6c^{low} monocytes, pDCs, and cDCs panel.

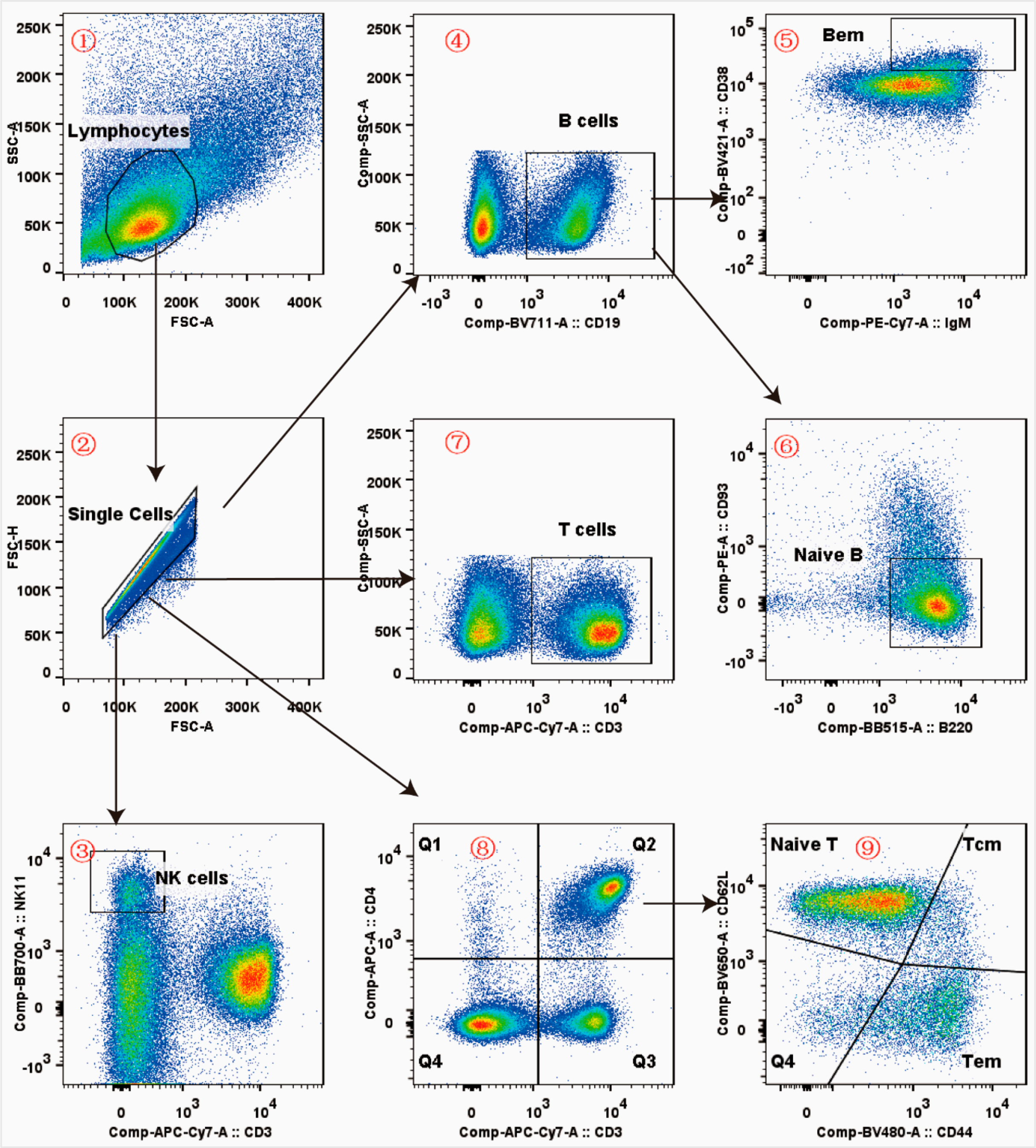

Supplementary Figure S3. Gating strategies for the lymphocytes, NK, T cells, Naïve T, Tem, B cells, Naïve B, and Bem panel.

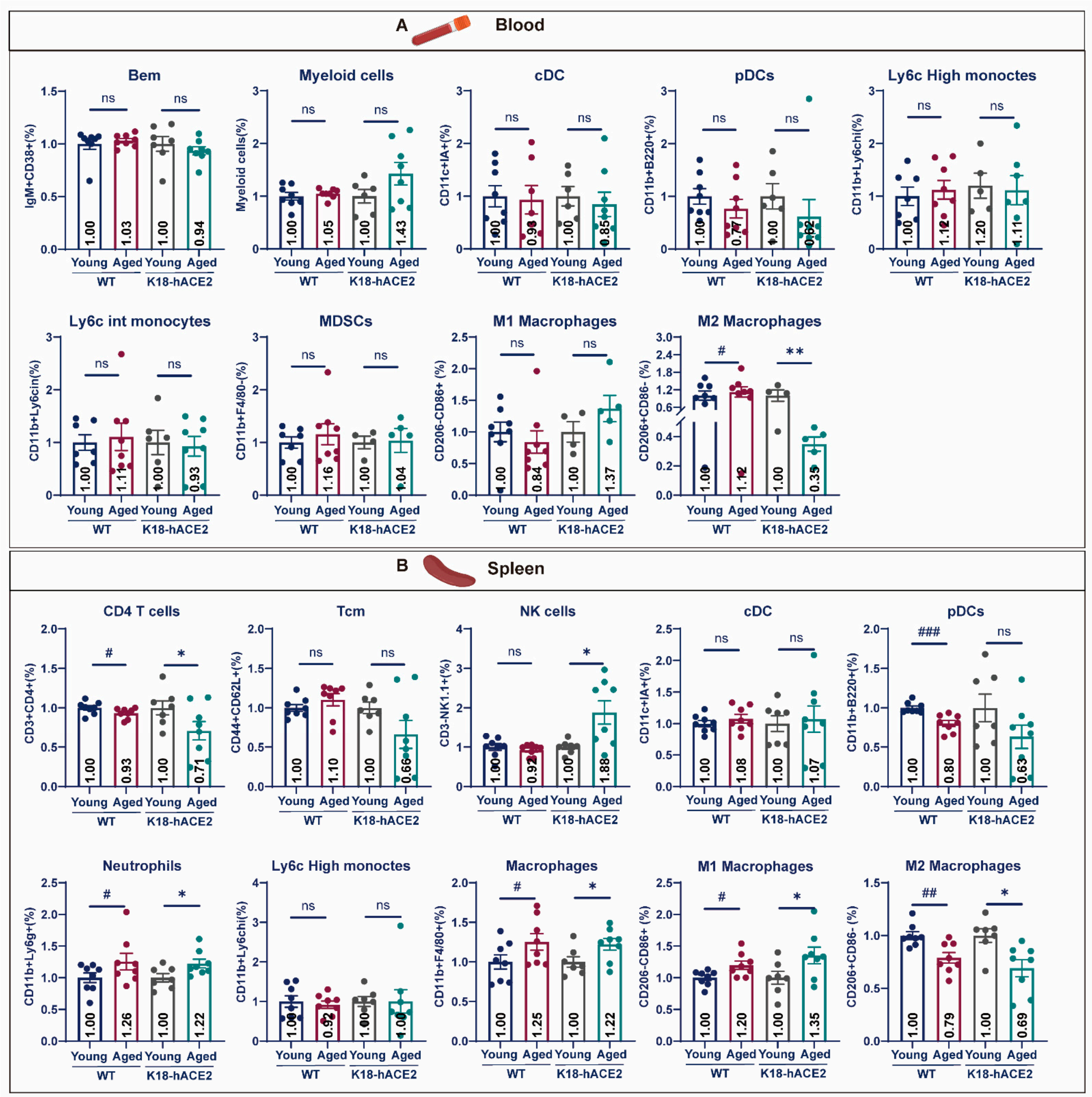

Supplementary Figure S4. A, Relative numbers of innate and adaptive immune cells in mouse spleen. **B,** Relative numbers of innate and adaptive immune cells in mouse blood. Results are expressed as mean ± SEM. #*P*<0.05, ##*P*<0.01 and ###*P*<0.001 versus the WT group. **P*<0.05 and ***P*<0.01 versus the K18-hACE2 group.

Gene	Forward primer	Reverse primer
hACE2	AAACATACTGTGACCCCGCAT	CCAAGCCTCAGCATATTGAACA
GAPDH	GTCCATGCCATCACTGCCACTC	CGCCTGCTTCACCACCTTCTTG

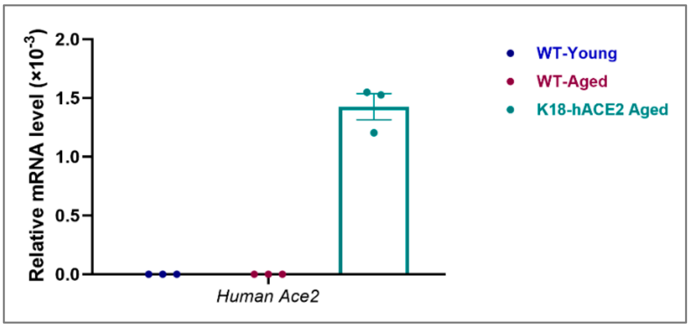

Supplementary Figure S5. Relative mRNA levels of human *ACE2* in heart (n=3). Results are expressed as mean ± SEM.

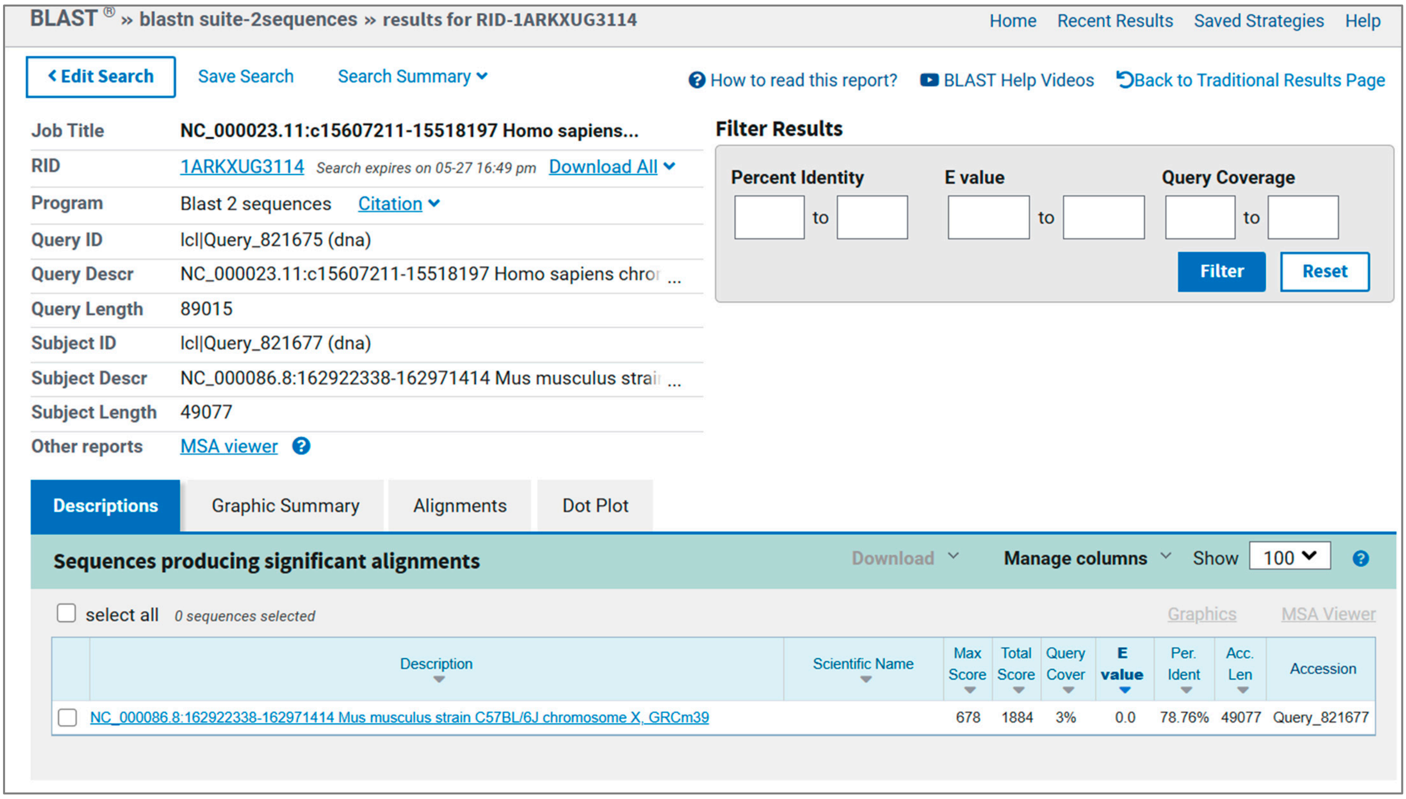

Supplementary Figure S6. Sequence alignment of human and mouse *Ace2*.
